# Mechanisms underlying superior outcomes of transcatheter aortic valve implantation with the latest balloon expandable valve

**DOI:** 10.1038/s44325-026-00105-w

**Published:** 2026-03-02

**Authors:** Juri Iwata, Masanori Yamamoto, Ryo Arita, Tomonari Moriizumi, Toshinobu Ryuzaki, Hikaru Tsuruta, Shinichi Shirai, Yusuke Watanabe, Toru Naganuma, Futoshi Yamanaka, Masahiko Noguchi, Hiroshi Ueno, Yohei Ohno, Masaki Izumo, Hidetaka Nishina, Masahiko Asami, Gaku Nakazawa, Fumiaki Yashima, Hirofumi Hioki, Tetsuro Shimura, Kenichi Ishizu, Toshiaki Otsuka, Hideyuki Shimizu, Masaki Ieda, Kentaro Hayashida

**Affiliations:** 1 Department of Cardiology, Keio University School of Medicine, Tokyo, Japan; 2 Department of Cardiology, Toyohashi Heart Center, Toyohashi, Japan; 3 Department of Cardiology, Nagoya Heart Center, Nagoya, Japan; 4 Department of Cardiology, Gifu Heart Center, Gifu, Japan; 5 Department of Cardiology, Kokura Memorial Hospital, Kokura, Japan; 6 Department of Cardiology, Teikyo University, Tokyo, Japan; 7 Department of Cardiology, New Tokyo Hospital, Chiba, Japan; 8 Department of Cardiology, Shonan Kamakura General Hospital, Kanagawa, Japan; 9 Department of Cardiology, Tokyo Bay Urayasu Ichikawa Medical Center, Urayasu, Japan; 10 Cardiology, Toyama University Hospital, Toyama, Japan; 11 Department of Cardiology, Tokai University, Isehara, Japan; 12 Department of Cardiology, St. Marianna University School of Medicine, Kawasaki, Japan; 13 Department of Cardiology, Tsukuba Medical Center Hospital, Tsukuba, Japan; 14 Division of Cardiology, Mitsui Memorial Hospital, Tokyo, Japan; 15 Department of Cardiology, Kindai University, Osaka, Japan; 16 Department of Cardiology, Saiseikai Utsunomiya Hospital, Utsunomiya, Japan; 17 Department of Cardiology, IMS Tokyo Katsushika General Hospital, Tokyo, Japan; 18 Department of Hygiene and Public Health, Nippon Medical School, Tokyo, Japan; 19 Department of Cardiovascular Surgery, Keio University School of Medicine, Tokyo, Japan

**Keywords:** Cardiology, Diseases, Medical research

## Abstract

Transcatheter aortic valve implantation (TAVI) using SAPIEN 3 Ultra RESILIA (S3UR) offers improved hemodynamic performance than its former generation, SAPIEN 3 (S3). This study compared 1-year clinical outcomes after TAVI using S3UR and S3. Among 2,369 patients from the OCEAN-TAVI registry (UMIN000020423), a 1:1 propensity score-matched analysis identified 775 matched pairs. One-year post-TAVI, S3UR showed significantly lower all-cause mortality (10.3% vs. 13.4%, *p* = 0.026), stroke (0.9% vs. 3.4%, *p* = 0.001), and heart failure rehospitalization (1.4% vs. 2.7%, *p* < 0.001) than S3. These differences were pronounced in patients receiving smaller valves (20–23 mm). S3UR demonstrated a larger effective orifice area, lower mean pressure gradient, and lower incidence of paravalvular leakage than S3 at discharge. At 1 year, S3UR showed significantly reduced paravalvular leakage and lower incidences of mean pressure gradient ≥20 mmHg than S3. We concluded that S3UR demonstrated superior hemodynamic performance to S3, exhibiting better prognosis, particularly in patients with smaller valves.

## Introduction

The excellent clinical outcomes of transcatheter aortic valve implantation (TAVI), especially with balloon-expandable (BE) transcatheter heart valves (THVs), are well established^1,2^. The SAPIEN 3 Ultra RESILIA (S3UR) is the fifth-generation valve in the SAPIEN series (Edwards Lifesciences, Irvine, CA, USA), the most commonly used BE-THV. Recent advancements in BE-THVs have led to significant clinical improvements, including reduced paravalvular leakage (PVL) and enhanced echocardiographic parameters of valve hemodynamics^3–5^. Additionally, surgical bioprosthetic valves incorporating RESILIA tissue have demonstrated similar favorable hemodynamic profiles on echocardiographic assessments^6^. S3UR, which exhibits more favorable post-procedural echocardiographic hemodynamics, was associated with improved 1-year clinical outcomes compared with the SAPIEN 3 (S3) or S3 Ultra^4^. However, whether these improvements in device performance translate to better clinical outcomes in more general populations remains to be broadly investigated. Herein, we assessed the 1-year clinical outcomes and hemodynamic performance of S3UR compared with those of S3, focusing on its impact on patients with small THVs and aortic annulus sizes, using data from a nationwide multicenter registry.

## Results

### Study population and baseline characteristics

**Fig. 1:**
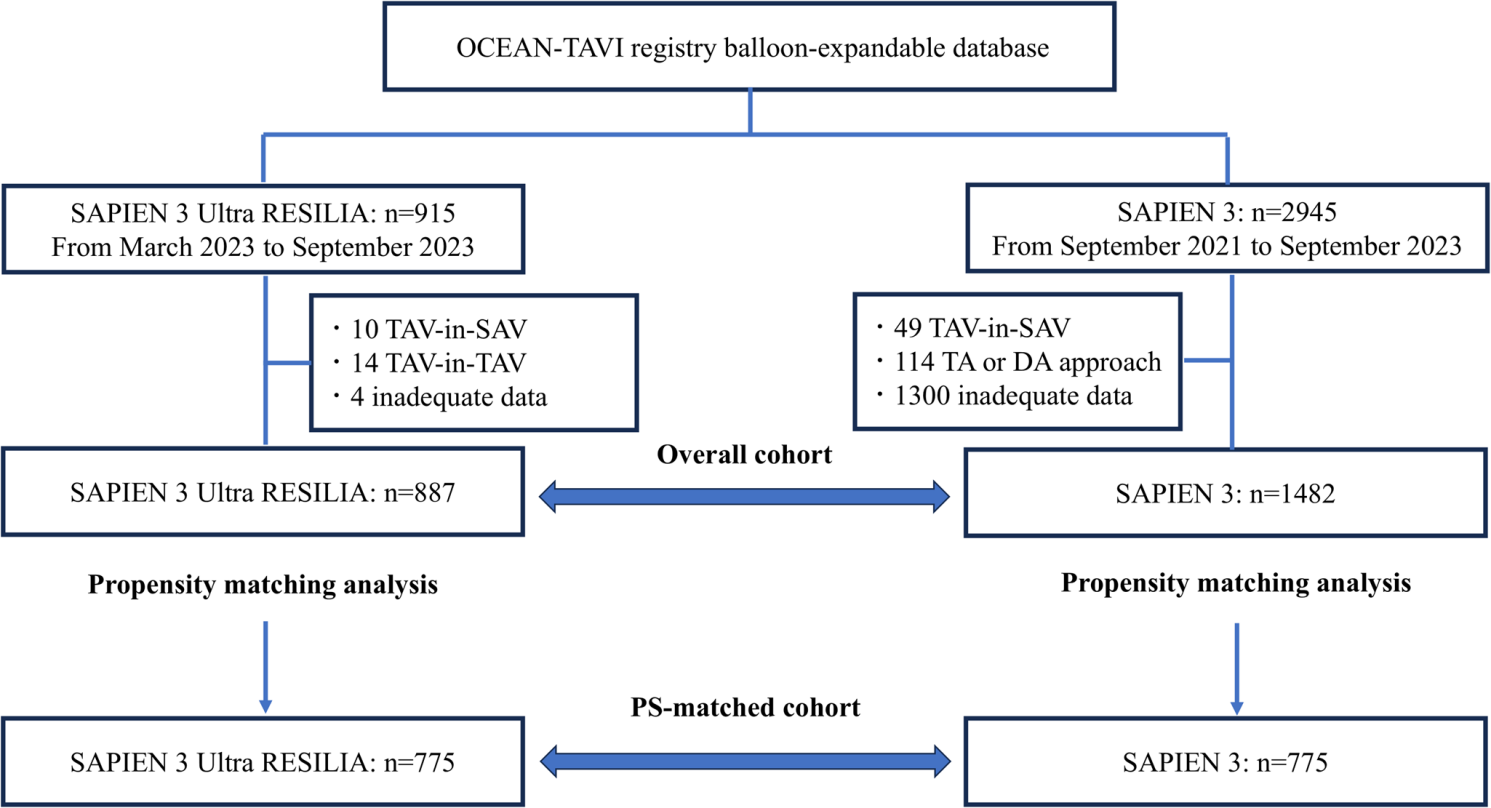
Study flow chart. OCEAN-TAVI Optimized transcatheter vAlvular iNtervention – Transcatheter Aortic Valve Implantation, TAV transcatheter aortic valve, SAV surgical aortic valve, TA transapical, DA direct aorta.

Among the 915 and 2945 patients who underwent TAVI with S3UR and S3, 887 and 1482 met the inclusion criteria, respectively, and were included herein. A 1:1 propensity score matching resulted in 775 patients each in the S3UR and S3 groups (Fig. 1). The baseline characteristics of the unmatched and matched cohorts are presented in Table 1. Before propensity score matching, the groups differed in several parameters, including clinical frailty scale scores and other comorbidities. In echocardiographic assessments, patients in the S3UR group exhibited a larger aortic valve area (0.69 ± 0.19 cm² vs. 0.67 ± 0.19 cm², *p* = 0.002) and higher left ventricular ejection fraction (59.3 ± 12.3% vs. 57.6 ± 13.4%, *p* = 0.004) compared with those in the S3 group. After propensity score matching, patient characteristics of the S3UR and S3 groups were well balanced, with absolute standardized differences <0.10 across all measured baseline characteristics.

**Table 1:** Baseline characteristics of unmatched and matched cohorts

	Pre-matched cohort			Matched cohort			
	S3UR	S3		S3UR	S3		
	*N* = 887	*N* = 1482	*p*	*N* = 775	*N* = 775	*p*	ASD
Age, years	83.8 ± 6.1	83.5 ± 6.3	0.177	83.6 ± 6.0	83.7 ± 6.4	0.485	0.085
Sex (male)	356 (40.1)	657 (44.3)	0.046	320 (41.3)	314 (40.5)	0.757	0.016
Body mass index, kg/m²	22.3 ± 3.9	22.2 ± 3.8	0.521	22.4 ± 3.9	22.4 ± 3.9	0.673	0.017
STS-PROM score, %	6.0 [4.0–9.1]	6.1 [3.8–9.5]	0.769	5.9 [4.0–8.9]	5.7 [3.7–9.4]	0.54	0
Clinical frailty scale ≥4	195 (22.0)	365 (24.6)	<0.001	175 (22.6)	188 (24.3)	0.516	0.041
NYHA III or IV	289 (32.6)	454 (30.6)	0.081	233 (30.1)	253 (32.6)	0.274	0.056
Hemoglobin, g/dL	11.5 [10.4–12.7]	11.4 [10.4–12.6]	0.572	11.5 [10.5–12.8]	11.5 [10.3–12.7]	0.385	0.055
Brain Natrium Peptide, pg/ml	194 [82–554]	312 [125–827]	<0.001	190 [80–534]	270 [110–705]	0.001	0.144
Concomitant diseases							
Hypertension	716 (80.7)	1183 (80.0)	0.687	626 (80.8)	648 (83.6)	0.144	0.025
Diabetes mellitus	296 (33.4)	456 (30.8)	0.101	251 (32.4)	238 (30.7)	0.477	0.036
Dyslipidemia	463 (52.2)	749 (50.7)	0.484	413 (53.3)	390 (50.4)	0.253	0.058
Chronic kidney disease (GFR < 60 mL/min/1.73 m²)	672 (75.8)	1141 (77.3)	0.407	582 (75.1)	586 (75.6)	0.814	0.012
Dialysis	181 (20.4)	411 (27.8)	<0.001	166 (21.4)	177 (22.8)	0.501	0.034
COPD	89 (10.0)	127 (8.6)	0.237	67 (8.6)	67 (8.6)	1	0
Atrial fibrillation	197 (22.2)	321 (21.8)	0.799	159 (20.5)	176 (22.7)	0.294	0.053
Coronary artery disease	293 (33.0)	545 (36.8)	0.065	262 (33.8)	263 (33.9)	0.957	0.003
Peripheral artery disease	142 (16.0)	261 (17.7)	0.298	119 (15.4)	121 (15.6)	0.888	0.007
Carotid stenosis	58 (6.5)	69 (4.7)	0.050	47 (6.1)	40 (5.2)	0.436	0.04
Previous history							
Smoking	246 (28.2)	462 (31.9)	0.064	213 (28.1)	242 (31.9)	0.104	0.083
History of PCI	158 (17.8)	333 (22.5)	0.006	143 (18.5)	167 (21.6)	0.124	0.078
History of CABG	32 (3.6)	56 (3.8)	0.831	28 (3.6)	22 (2.8)	0.388	0.044
History of myocardial infarction	47 (5.3)	119 (8.0)	0.011	43 (5.5)	55 (7.1)	0.21	0.064
History of stroke	91 (10.3)	194 (13.1)	0.022	85 (11.0)	90 (11.6)	0.561	0.02
Previous pacemaker	47 (5.3)	84 (5.7)	0.149	40 (5.2)	41 (5.3)	0.909	0.034
Echocardiographic data				1.148	0.968–1.360	0.113	
Aortic valve area, cm²	0.69 ± 0.19	0.67 ± 0.19	0.002	0.69 ± 0.19	0.68 ± 0.19	0.292	0.035
Index aortic valve area, cm²/m²	0.47 ± 0.13	0.45 ± 0.13	<0.001	0.47 ± 0.13	0.46 ± 0.13	0.406	0.028
Peak flow velocity, m/s	4.3 ± 0.65	4.3 ± 0.71	0.989	4.3 ± 0.65	4.3 ± 0.68	0.628	0.013
Aortic valve peak gradient, mmHg	74.9 ± 22.6	75.1 ± 25.2	0.922	74.4 ± 22.7	74.7 ± 23.7	0.693	0.012
Aortic valve mean gradient, mmHg	43.3 ± 14.4	43.5 ± 15.6	0.692	43.1 ± 14.4	43.4 ± 14.7	0.335	0.018
Left ventricular ejection fraction, %	59.3 ± 12.3	57.6 ± 13.4	0.004	59.4 ± 12.3	59.3 ± 12.3	0.594	0.011
Preserved ( ≥ 50%)	716 (80.7)	1121 (75.6)	0.005	623 (80.4)	630 (81.3)	0.865	0.016
Mid-range ( ≥ 40, <50%)	86 (9.7)	166 (11.2)		77 (9.9)	71 (9.2)		
Reduced ( < 40%)	85 (9.6%)	186 (12.6)		75 (9.7)	74 (9.5)		
Aortic regurgitation ≥moderate	109 (12.3)	197 (13.3)	0.037	86 (11.1)	86 (11.1)	1	0
Mitral regurgitation ≥moderate	93 (10.5)	305 (20.6)	<0.001	91 (11.7)	95 (12.3)	0.755	0.016
Tricuspid regurgitation ≥moderate	58 (6.5)	164 (11.1)	<0.001	50 (6.5)	56 (7.2)	0.546	0.031
Computed Tomography							
Annulus Area, mm^2^	424 ± 78	420 ± 74	0.426	423 ± 76	421 ± 77	0.572	0.026
Area <430 mm^2^, %	523 (59.0)	886 (59.8)	0.006	456 (58.8)	488 (63.0)	0.096	
Perimeter, mm	74.4 ± 6.8	74.0 ± 6.6	0.117	74.2 ± 6.6	73.3 ± 6.7	0.444	0.032

Data are depicted as means with standard deviations (±SD) or counts with percentages (%).

*S3UR* SAPIEN 3 Ultra RESILIA, *S3* SAPIEN 3, *STS-PROM* Society of Thoracic Surgeons-predicted risk of mortality, *NYHA* New York Heart Association, *GFR* Glomerular Filtration Rate, *COPD* Chronic Obstructive Pulmonary Disease, *PCI* percutaneous coronary intervention, *CABG* coronary artery bypass grafting, *ASD* absolute standardized difference.

*P*-values are from Fisher’s test (2 × 2 comparison), chi-squared test (*n* x 2 comparisons), or *t*-tests (continuous parameters).

### Procedural characteristics, complications, and echocardiographic outcomes

**Fig. 2:**
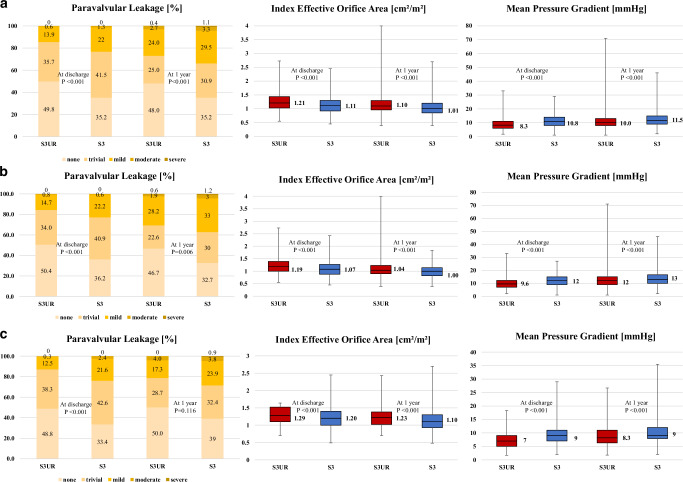
Echocardiographic data at discharge and 1 year after transcatheter aortic valve implantation comparing the S3UR and S3 valves. S3UR, SAPIEN 3 Ultra RESILIA; S3, SAPIEN 3. **a** Overall (**b**) Small valve size (20 mm or 23 mm) (**c**) Large valve size (26 mm or 29 mm)

The procedures, complications, and echocardiographic outcomes are shown in Table 2. After matching, no significant differences were observed in the incidence of procedural complications, including in-hospital death, stroke, or conversion to open surgery, between the S3UR and S3 groups (all *p* > 0.05). Post-procedural echocardiography revealed a larger EOA (1.88 ± 0.49 cm^2^ vs. 1.68 ± 0.45 cm^2^), lower peak flow velocity (2.0 ± 0.45 m/s vs. 2.3 ± 0.45 m/s), and lower mPG (9.0 ± 4.1 mmHg vs. 11.3 ± 4.5 mmHg) in the S3UR group compared with the S3 group (all *p* < 0.001, Fig. 2a). Patients in the S3UR group had a lower incidence of PPM (moderate: 46 [5.9%] vs. 84 [10.8%], severe: 3 [0.4%] vs. 24 [3.1%]) and a lower incidence of PVL (none: 384 [49.8%] vs. 270 [35.2%], trivial: 275 [35.7%] vs. 319 [41.5%], mild: 107 [13.9%] vs. 169 [22.0%], moderate: 5 [0.6%] vs. 10 [1.3%], severe: 0% vs. 0%) than those in the S3 group (all *p* < 0.001). The prevalence of NYHA III or IV was significantly lower in the S3UR group than in the S3 group (11 [2.5%] vs. 20 [5.0%], *p* < 0.001). These results were similar for both small and large valve sizes (Fig. 2b,c).

**Table 2:** Procedure, complications, and echocardiographic outcomes

	Prematched cohort: overall			Matched cohort: overall				Matched: small (20 mm or 23 mm)			Matched: large (26 mm or 29 mm)		
	S3UR	S3		S3UR	S3			S3UR	S3		S3UR	S3	
	*N* = 887	*N* = 1482	*p*	*N* = 775	*N* = 775	*p*	ASD	*N* = 471	*N* = 477	*p*	*N* = 304	*N* = 298	p
Transfemoral	844 (95.2)	1420 (95.8)	0.447	739 (95.4)	741 (95.6)	0.807	0.012	491 (96.5)	488 (96.4)	0.986	302 (93.8)	306 (94.2)	0.845
**Valve size**													
20 mm	70 (7.9)	73 (4.9)	0.003	53 (6.8)	56 (7.2)	0.977	0.012	53 (11.3)	56 (11.7)	0.814			
23 mm	476 (53.7)	775 (52.3)		418 (53.9)	421 (54.3)			418 (88.7)	421 (88.3)				
26 mm	276 (31.1)	540 (36.4)		250 (32.3)	243 (31.4)						250 (82.2)	243 (81.5)	0.825
29 mm	65 (7.3)	94 (6.3)		54 (7.0)	55 (7.1)						54 (17.8)	55 (18.5)	
Local anesthesia	651 (73.4)	1009 (68.1)	0.012	569 (73.4)	507 (65.4)	<0.001	0.174	345 (73.7)	303 (63.5)	0.001	224 (73.7)	204 (68.5)	0.157
Pre-dilatation	141 (15.9)	196 (13.3)	0.077	115 (14.8)	124 (16.0)	0.527	0.032	72 (15.3)	88 (18.4)	0.194	43 (14.1)	36 (12.1)	0.453
Post-dilatation	101 (11.4)	260 (17.6)	<0.001	94 (12.1)	111 (14.3)	0.202	0.065	68 (14.4)	74 (15.5)	0.642	26 (8.6)	37 (12.4)	0.122
**Echocardiographic data**													
Effective orifice area, cm²	1.87 ± 0.49	1.67 ± 0.47	<0.001	1.88 ± 0.49	1.68 ± 0.45	<0.001		1.73 ± 0.42	1.52 ± 0.37	<0.001	2.12 ± 0.50	1.92 ± 0.45	<0.001
Index effective orifice area, cm²/m²	1.27 ± 0.33	1.13 ± 0.30	<0.001	1.27 ± 0.33	1.13 ± 0.29	<0.001		1.23 ± 0.32	1.08 ± 0.27	<0.001	1.34 ± 0.34	1.22 ± 0.30	<0.001
Peak flow velocity, m/s	2.0 ± 0.45	2.3 ± 0.44	<0.001	2.0 ± 0.45	2.3 ± 0.45	<0.001		2.2 ± 0.44	2.4 ± 0.44	<0.001	1.9 ± 0.38	2.1 ± 0.40	<0.001
Mean pressure gradient, mmHg	9.0 ± 4.2	11.4 ± 4.6	<0.001	9.0 ± 4.1	11.3 ± 4.5	<0.001		10.2 ± 4.3	12.5 ± 4.6	<0.001	7.3 ± 3.0	9.4 ± 3.8	<0.001
Mean pressure gradient ≥20 mmHg	20 (2.3)	78 (5.3)	<0.001	16 (2.1)	39 (5.0)	0.002		16 (3.4)	33 (6.9)	0.034	0 (0)	6 (2.0)	0.017
Prosthesis-patient mismatch													
none	820 (92.4)	1199 (80.9)	<0.001	718 (92.6)	644 (83.1)	<0.001		428 (90.9)	376 (81.7)	<0.001	290 (96.3)	268 (91.8)	0.047
moderate	55 (6.2)	167 (11.3)		46 (5.9)	84 (10.8)			35 (7.4)	63 (13.7)		11 (3.7)	21 (7.2)	
severe	3 (0.3)	52 (3.5)		3 (0.4)	24 (3.1)			3 (0.6)	21 (4.6)		0 (0)	3 (1.0)	
Paravalvular leakage ≥mild	126 (14.3)	308 (20.8)	<0.001	112 (14.5)	179 (23.1)	<0.001		73 (15.5)	108 (22.9)	0.014	39 (12.8)	71 (24.0)	0.002
Mitral regurgitation ≥moderate	70 (7.9)	189 (12.8)	<0.001	61 (7.9)	66 (8.5)	0.754		35 (7.5)	42 (8.9)	0.574	26 (8.6)	24 (8.1)	0.976
Tricuspid regurgitation ≥moderate	79 (9.0)	144 (9.7)	0.012	66 (8.5)	45 (5.8)	0.054		40 (8.5)	31 (6.6)	0.322	26 (8.6)	14 (4.9)	0.12
Ent days	6 [4–9]	6 [4–8]	0.400	6 [4–9]	6 [4–8]	0.702		5 [4–9]	6 [4–8]	0.382	6 [4–9]	6 [4–8]	0.636
In-hospital death	12 (1.4)	32 (2.2)	0.159	12 (1.5)	17 (2.2)	0.349		8 (1.7)	15 (3.1)	0.148	4 (1.3)	2 (0.7)	0.426
Open surgery	0 (0)	1 (0.1)	0.122	0 (0)	1 (0.1)	0.134		0 (0)	1 (0.2)	0.226	0 (0)	0 (0)	NA
Life-threatening and major bleeding	57 (6.4)	99 (6.7)	0.809	43 (5.5)	50 (6.5)	0.454		22 (4.7)	33 (6.9)	0.139	21 (6.9)	17 (5.7)	0.544
Disabling stroke	6 (0.7)	15 (1.0)	0.399	6 (0.8)	10 (1.3)	0.315		4 (0.8)	8 (1.7)	0.254	2 (0.7)	2 (0.7)	0.984
Major vascular complication	14 (1.6)	26 (1.8)	0.748	11 (1.4)	15 (1.9)	0.429		6 (1.3)	11 (2.3)	0.231	5 (1.6)	4 (1.3)	0.76
Tamponade	2 (0.2)	3 (0.2)	0.970	2 (0.3)	2 (0.3)	0.914		2 (0.4)	2 (0.5)	0.94	0 (0)	0 (0)	NA
New pacemaker implantation	45 (5.1)	77 (5.2)	0.220	40 (5.2)	44 (5.1)	0.157		20 (4.2)	26 (5.4)	0.205	20 (6.6)	14 (4.7)	0.33
Discharge NYHA III or IV	17 (2.0)	52 (4.0)	0.011	14 (1.8)	28 (3.6)	<0.001		11 (2.5)	20 (5.0)	<0.001	3 (1.0)	8 (3.0)	0.003

Data are depicted as means with standard deviations (±SD) or counts with percentages (%).

*S3UR* SAPIEN 3 Ultra RESILIA, *S3* SAPIEN 3, *NYHA* New York Heart Association, *ASD* absolute standardized difference.

*P*-values are from Fisher’s test (2 × 2 comparison), chi-squared test (*n* x 2 comparisons), or t-tests (continuous parameters).

Follow-up echocardiographic and other clinical data at 1 year, available for 1097 of the 1291 living patients (85.0%), are summarized in Table 3. At the 1-year follow-up, patients in the S3UR group showed significantly better echocardiographic parameters than those in the S3 group (EOA: 1.70 ± 0.45 cm^2^ vs. 1.56 ± 0.44 cm^2^; peak flow velocity: 2.3 ± 0.48 m/sec, vs 2.4 ± 0.48 m/sec; mPG: 11.1 ± 5.5 mmHg vs. 12.5 ± 5.5 mmHg) (all *p* < 0.001, Fig. 2a). These results were similar for small and large valves in both groups (Fig. 2b, c). At discharge, the incidence of PVL ≥mild was significantly lower in the S3UR group than in the S3 group and 1 year after TAVI, the overall incidence of PVL ≥mild remained significantly lower in S3UR than in S3 (142 [27.1%] vs. 184 [33.9%], *p* = 0.032). However, when analyzed by valve size, no significant difference was observed in either the small or large sizes (small: 99 [30.7%] vs. 123 [37.2%], *p* = 0.199; large: 43 [21.3%] vs. 61 [28.6%], *p* = 0.093). The proportion of patients whose PVL grade increased by one or more points 1 year after TAVI than at discharge was comparable between the S3UR and S3 groups (143 [27.3%] vs. 174 [32.2%], *p* = 0.086). The number of patients with mPG ≥20 mmHg was significantly lower in the S3UR group than in the S3 group for smaller valves; however, no significant differences between the groups were observed for larger valves (small valve sizes: 22 [6.6%] vs. 47 [14.2%], *p* = 0.007; large valve sizes: 43 [21.3%] vs. 61 [28.6%], *p* = 0.093).

**Table 3:** One-year outcomes

	Overall			Small (20 mm or 23 mm)			Large (26 mm or 29 mm)		
	Matched cohort								
	S3UR	S3		S3UR	S3		S3UR	S3	
			*p*			*p*			*p*
Left ventricular ejection fraction, %	63.4 ± 8.1	63.2 ± 9.3	0.429	65.2 ± 6.6	64.9 ± 7.8	0.782	60.8 ± 9.4	60.6 ± 10.8	0.285
Effective orifice area, cm²	1.70 ± 0.45	1.56 ± 0.44	<0.001	1.53 ± 0.39	1.41 ± 0.35	<0.001	1.97 ± 0.42	1.80 ± 0.47	<0.001
Index effective orifice area, cm²/m²	1.15 ± 0.32	1.06 ± 0.30	<0.001	1.09 ± 0.33	1.00 ± 0.27	<0.001	1.24 ± 0.29	1.13 ± 0.32	<0.001
Peak flow velocity, m/s	2.3 ± 0.48	2.4 ± 0.48	<0.001	2.4 ± 0.48	2.5 ± 0.47	<0.001	2.0 ± 0.40	2.2 ± 0.41	<0.001
Mean pressure gradient, mmHg	11.1 ± 5.5	12.5 ± 5.5	<0.001	12.5 ± 5.9	14.0 ± 5.7	<0.001	8.8 ± 3.8	10.1 ± 4.2	<0.001
Mean pressure gradient ≥20 mmHg	25 (3.2)	52 (6.7)	0.006	22 (6.6)	47 (14.2)	0.007	3 (1.4)	5 (2.2)	0.237
Prosthesis-patient mismatch									
none	439 (85.9)	403 (75.5)	<0.001	261 (82.1)	229 (70.5)	0.003	178 (92.2)	174 (83.3)	0.006
moderate	59 (11.5)	101 (18.9)		44 (13.8)	73 (22.5)		15 (7.8)	28 (13.4)	
severe	13 (2.5)	30 (5.6)		13 (4.1)	23 (7.1)		0 (0)	7 (3.3)	
Paravalvular leakage ≥ mild	142 (27.1)	184 (33.9)	0.032	99 (30.7)	123 (37.2)	0.199	43 (21.3)	61 (28.6)	0.093
Increased paravalvular leakage	143 (27.3)	174 (32.2)	0.086	95 (29.5)	121 (36.7)	0.052	48 (23.9)	53 (25.1)	0.770
Mitral regurgitation ≥moderate	41 (7.8)	42 (7.7)	0.711	26 (7.9)	29 (8.8)	0.925	15 (7.5)	13 (6.1)	0.314
Tricuspid regurgitation ≥moderate	51 (9.6)	52 (9.6)	0.711	27 (8.3)	34 (10.2)	0.679	24 (11.9)	18 (8.5)	0.241
NYHA III or IV	5 (1.0)	14 (2.7)	0.040	4 (1.3)	8 (2.5)	0.248	1 (0.5)	6 (2.9)	0.061
Brain Natrium Peptide, pg/ml	93 [46-189]	111 [52–253]	0.040	94 [46–187]	118 [57–264]	0.018	93 [47–211]	101 [48–237]	0.706

Data are depicted as means with standard deviations ( ± SD) or counts with percentages (%).

*S3UR* SAPIEN 3 Ultra RESILIA, *S3* SAPIEN 3, *NYHA* New York Heart Association.

*P*-values are from Fisher’s test (2 × 2 comparison), chi-squared test (*n* x 2 comparisons), or *t*-tests (continuous parameters).

### One-year clinical outcomes

**Fig. 3:**
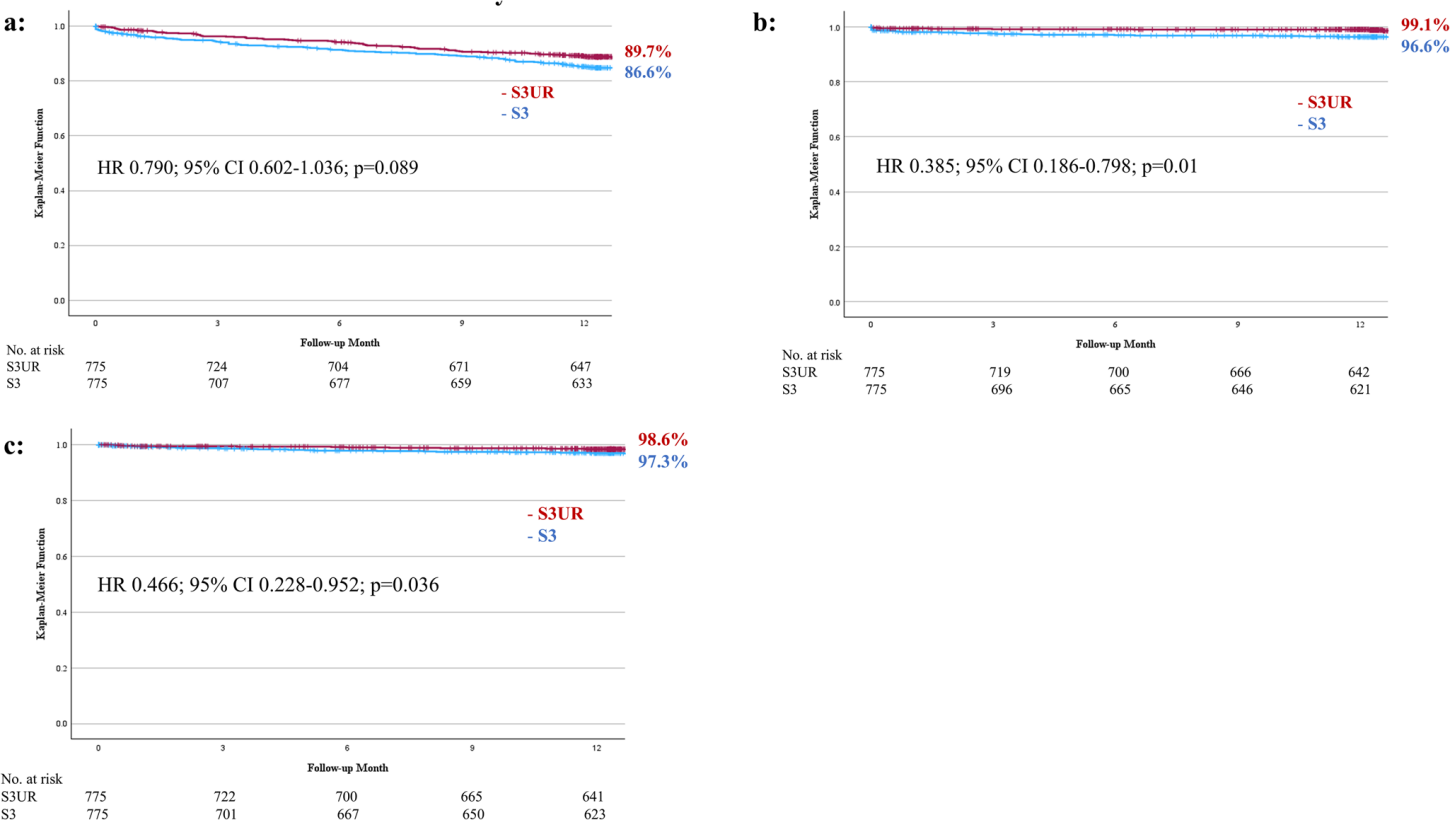
Kaplan–Meier curves comparing 1-year clinical outcomes for S3UR and S3 valves. **a** All-cause mortality (**b**) Stroke (**c**) Heart failure rehospitalization. S3UR SAPIEN 3 Ultra RESILIA, S3 SAPIEN 3, HR hazard ratio, CI confidence interval.

The average follow-up duration for living patients was 469 ± 260 days. Kaplan–Meier curves depicting the clinical outcomes according to the groups are shown in Fig. 3 and Table 4. Throughout the study period, 1-year mortality occurred in 10.3% and 13.4% of patients in the S3UR and S3 groups (*p* = 0.026), respectively, which tended to be lower in the S3UR group than in the S3 (HR 0.790; 95% CI 0.602–1.036; *p* = 0.089). Additionally, the incidence of stroke and HFH at 1 year was significantly lower in the S3UR group than in the S3 group, respectively (stroke; 0.9 vs. 3.4%, *p* = 0.001, HR 0.385; 95% CI 0.186–0.798; *p* = 0.01) (HFH; 1.4% vs. 2.7%, *p* < 0.001, HR 0.665; 95% CI 0.228–0.952; *p* = 0.036). None of the patients underwent aortic valve reintervention during the follow-up period. In S3UR, the rate of new pacemaker implantation excluding the perioperative incidence was 0%, and in S3, it was 3/775 (0.004%). We did not collect the data precisely divided into cardiovascular and non-cardiovascular mortality in this study. In the analyses according to the valve size, 1-year mortality was significantly lower in the S3UR group than in the S3 group for smaller valve sizes (9.7% vs. 14.7%, *p* = 0.011, HR 0.686; 95% CI 0.483–0.975; *p* = 0.036) but not for larger valve sizes (10.8% vs. 11.2%, *p* = 0.734, HR 0.984; 95% CI 0.638–1.516; *p* = 0.940). Additionally, the incidence of stroke and HFH was significantly lower in the S3UR group than in the S3 group respectively for smaller valve sizes (stroke; 0.9% vs. 4.2%, *p* = 0.003, HR 0.359; 95% CI 0.152–0.851; *p* = 0.02, HFH; 1.4% vs. 3.0%, *p* < 0.001, HR 0.366; 95% CI 0.144–0.932; *p* = 0.035) but not for larger valve sizes (stroke; 1.0% vs. 2.3%, *p* = 0.191, HR 0.473; 95% CI 0.119–1.872; *p* = 0.286, HFH; 1.4% vs. 2.2%, *p* = 0.191, HR 0.686; 95% CI 0.221–2.128; *p* = 0.514).

**Table 4:** One-year clinical outcomes

	Overall					Small (20 mm or 23 mm)					Large (26 mm or 29 mm)				
	Matched cohort														
	S3UR	S3	HR	95% CI	*p*	S3UR	S3	HR	95% CI	*p*	S3UR	S3	HR	95% CI	p
All-cause mortality	77 (10.3)	100 (13.4)	0.79	0.602–1.036	0.089	44 (9.7)	68 (14.7)	0.686	0.483–0.975	0.036	33 (9.8)	32 (11.2)	0.984	0.638–1.516	0.94
Stroke	7 (0.9)	25 (3.4)	0.385	0.186–0.798	0.01	4 (0.9)	19 (4.2)	0.359	0.152–0.851	0.02	3 (1.0)	6 (2.3)	0.473	0.119–1.872	0.286
Heart failure rehospitalization	10 (1.4)	19 (2.7)	0.466	0.228–0.952	0.036	6 (1.4)	13 (3.0)	0.366	0.144–0.932	0.035	4 (1.4)	6 (2.2)	0.686	0.221–2.128	0.514

Cumulative even rate (%).

*S3UR* SAPIEN 3 Ultra RESILIA, *S3* SAPIEN 3, *HR* Hazard ratio, *CI* Confidence interval.

*P*-values, HR, and 95% CI from the COX proportional hazard model.

**Fig. 4:**
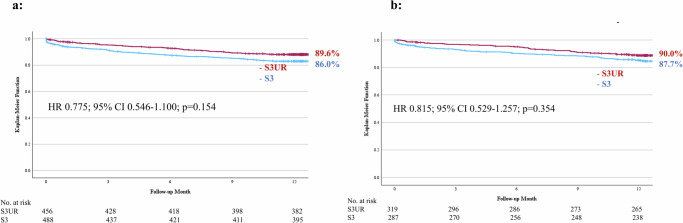
Kaplan–Meier curves comparing 1-year clinical outcomes for annulus areas <430 mm² and ≥430 mm². **a** Area <430 mm² (**b**) Area ≥430 mm². HR hazard ratio, CI confidence interval.

We also compared the all-cause mortality in the S3UR and S3 groups according to the annulus size: <430 mm² and ≥430 mm²; however, no significant difference in all-cause mortality between the groups was observed (<430 mm²: HR 0.775; 95% CI 0.546–1.100; *p* = 0.154, ≥430 mm²: HR 0.815; 95% CI 0.529–1.257; *p* = 0.354) (Fig. 4).

## Discussion

The salient findings of the present study are as follows. (1) S3UR, the latest generation BE-THV, demonstrated better hemodynamic parameters in echocardiography than S3, both at discharge and 1 year after TAVI. (2) Overall, mortality, stroke, and HFH were significantly lower in the S3UR group than in the S3 group during the 1-year follow-up. These differences were more prominent in patients with smaller valve sizes, whereas the outcomes were comparable in those with larger valve sizes. (3) At discharge, the rate of PVL was significantly lower in the S3UR group than in the S3 group; however, it increased over 1 year regardless of the valve size.

Previous studies have reported that S3UR valves demonstrate better hemodynamic performance in echocardiography than the former generations of BE-THVs^4,5,7^. The novelty of this study lies in making the most of this registry, which includes a relatively high proportion of small annuli, to analyze one-year outcomes and hemodynamics separately for small and large sizes. In the era of expanding indications for TAVI for younger and lower-risk patients, clarifying the developmental processes of appropriate treatment strategies is crucial to maximize the advantages of THVs. This study showed that the better early-phase echocardiographic parameters of S3UR　compared with those of S3, were maintained even 1 year after TAVI. These findings were consistent for both small and large valves. Notably, the lower incidence of mPG ≥20 mmHg at 1 year after TAVI in the S3UR group was statistically significant for smaller valves but not for larger ones. One of the reasons why clinical outcomes were superior in S3UR than S3 may be that S3UR demonstrated better echocardiographic hemodynamics than S3. Echocardiographic parameters were particularly favorable for smaller valve sizes. This is explained by the fact that the unique modification of the S3UR also changed the sewing maneuvers for each of the 3 leaflets at the commissural positions, especially for the smaller 20 mm and 23 mm valve sizes.

Regarding the higher incidence of stroke in S3 than in S3UR, one reason is that S3 is a historical cohort compared to S3UR, suggesting procedural inexperience and higher surgical risk in patient backgrounds. Furthermore, early intervention for aortic stenosis has the potential to reduce stroke rates long-term after TAVI. The widespread adoption of TAVI in Japan may make it easier for patients to undergo TAVI in S3UR compared to the S3 era.

Regarding PVL after TAVI, the current study demonstrated that S3UR exhibited a lower PVL rate than S3 at discharge. Our registry previously reported that S3UR was associated with lesser PVL than the latest self-expandable THVs, suggesting one of the pivotal advantages of the S3UR^8^. These combined results suggest a potential association between S3UR and improved prognosis, highlighting the remarkable changes in hemodynamic parameters. Recent data from the large-scale Transcatheter Valve Therapy Registry indicated improved 1-year outcomes for S3UR compared with earlier-generation BE-THVs^4^. Therein, the authors attributed the survival benefit primarily to the reduced incidence of mild PVL, in addition to improved hemodynamics. Some previous studies reported that patients with mild PVL had a significantly increased risk of mortality at 1 or more years after TAVI^9–11^. These results confirmed that minimizing PVL severity to less than mild is clinically important. However, PVL progression occurred in approximately 30% of patients in this cohort. Although the S3UR showed a lower incidence of PVL at discharge, this advantage appeared to be attenuated by 1 year. These trends were recognized in the previous Transcatheter Valve Therapy Registry data^4^. In this regard, this study showed that the S3UR group had significantly lower PVL than the S3 group 1 year after TAVI for smaller valves but not for larger ones. The mechanism of worsening PVL in patients undergoing TAVI with S3UR and S3 valves remains unclear. We speculate that one of the mechanisms may be related to the recoil of BE-THVs. The finding that this PVL difference was no longer significant one year after TAVI when we analyzed by valve sizes, suggests that the superior prognosis in S3UR than S3 may have been explained not by improvement in PVL at discharge alone. In any case, given that PVL increased over time and that more than mild PVL was associated with poorer prognosis of patients receiving BE-THVs, further long-term follow-up investigations are needed to elucidate future treatment strategies using S3UR.

Another contributing factor to the better clinical outcomes in the S3UR group may be the significantly lower incidence of PPM in this group. Although the clinical impact of moderate PPM remains inconclusive^12^, severe PPM is considered to be significantly associated with adverse prognosis, especially in patients with a small anatomy^13^. Patients with smaller annulus sizes and those who underwent TAVI with smaller valves, which are often used in those with a smaller body size, are well-known risk factors for PPM after TAVI^14,15^. Despite these anatomical characteristics, the incidence of PPM in this cohort was relatively low, even among patients with smaller valve sizes. Moreover, data from patients who underwent surgical aortic valve replacement revealed better post-procedural hemodynamics with the RESILIA tissue bioprosthesis than with the previous one^16,17^. The 20 mm and 23 mm S3UR valves incorporate a specific design in which each of the three leaflets is individually sewn at the commissural positions to allow for a larger EOA. Although better hemodynamics were observed for the 26 mm and 29 mm S3UR valves, the modifications in the 20 mm and 23 mm S3UR valves may contribute to better hemodynamics compared with previous models. Herein, an important new finding was that S3UR showed superior prognoses compared with S3 for smaller valve sizes but not for larger valve sizes. Some previous papers revealed that the mPG or PG ≥ 20 mmHg was not associated with prognosis^14^. In this study as well, a lower mPG of S3UR itself may not directly lead to improved prognosis. However, the multiple design improvements for 20 mm and 23 mm S3UR valves may have contributed to the enlargement of the indexed EOA, resulting in a lower incidence of severe PPM, fewer cases of mPG ≥20 mmHg, and lower PVL at 1 year after TAVI. These combined improvements in valve performance may have contributed to the improvement in prognosis. Furthermore, given that S3 represents a historical cohort compared to S3UR, we cannot rule out and describe in the limitation section the possibility that learning curves, or changes in anesthesia, may have influenced outcomes.

We speculate that one of the reasons for the favorable clinical outcomes in S3UR may be that the proportion of general anesthesia remained higher in S3 even after PSM. Previous studies have shown that patients who underwent TAVI with general anesthesia demonstrated worse outcomes, including mortality during short- and long-term follow-up, than those with local anesthesia^18,19^. While this finding is not definitive, it cannot be denied that the higher incidence of general anesthesia in S3 may have contributed to the poorer prognosis than S3UR.

This study highlights that the prognostic advantages of newer-generation BE-THVs extend to a broader population, supporting their broader applicability across diverse clinical settings. These findings provide meaningful insights into the ongoing debate regarding THV selection in patients with smaller anatomies.

Due to the use of observational, unblinded, and non-randomized registry data, this study has several limitations. First, as an observational cohort study, our findings may have been influenced by unmeasured variables and should be interpreted with caution. PSM is a useful statistical method for adjusting for confounding factors, but unlike randomized controlled trials, the potential for bias due to confounding factors could not be eliminated. Second, echocardiographic data were systematically collected and evaluated using the VARC-3 criteria; however, no independent core laboratories were included in this registry. Third, data from multi-detector computed tomography after TAVI were not available; thus, the phenomenon of decreasing EOA and increasing PVL could not be investigated in detail. Fourth, S3 is the valve historically used before the introduction of S3UR, and we do not have detailed data on inter-hospital and individual operator variations. Therefore, a learning curve may exist, and it is possible that the performance of S3, which was used earlier, may have impacted on some clinical outcomes. However, we used a database with a large number of cases and performed PSM, successfully matching patient backgrounds. The SAPIEN 3 Ultra (S3U) valve represented an improvement over the S3 valve, with its sealing skirt, which resulted in a reduction of the incidence of mild PVL. However, in the OCEAN-TAVI registry, the S3U valve was not reimbursed, and the next valve after the S3 was the S3UR. A key limitation of this study is that we were unable to determine whether the favorable outcomes resulted from RESILIA tissue itself or from improvements made to the skirt design starting at the S3U stage. Finally, while our cohort reflects the general population that undergoes TAVI in Japan, with a mean age of 85 years, these results may not be generalizable to younger populations with longer life expectancies and greater exercise capacity. However, the registry used herein had high-quality follow-up data, with more than 80% of patients undergoing 1-year echocardiography, enabling us to demonstrate reasonable results.

In conclusion, the S3UR valve demonstrated superior hemodynamic performance compared with the S3 valve 1 year after TAVI, exhibiting lower rates of all-cause mortality and composite adverse outcomes, particularly in patients with smaller valve sizes. Similar findings have previously been reported in Western populations, and the consistency of these results in an Asian cohort, despite notable differences in body size and ethnicity, corroborates the generalizability of the benefits associated with newer-generation BE-THVs.

## Methods

### Study cohort

The Optimized transCathEter vAlvular intervention-Transcatheter Aortic Valve Implantation (OCEAN-TAVI) registry is an ongoing nationwide prospective multicenter cohort study involving patients with aortic stenosis undergoing TAVI at 22 collaborating centers across Japan. Participating centers were mandated to enroll patients in the registry, adhere to the registry protocol, and provide patient information and clinical outcomes during follow-up. The ethics committee of each center approved the study protocol: Institutional Review Board (IRB) in Keio University School of Medicine, IRB in Toyohashi Heart Center, IRB in Nagoya Heart Center, IRB in Gifu Heart Center, IRB in Kokura Memorial Hospital, IRB in Teikyo University, IRB New Tokyo Hospital, IRB in Shonan Kamakura General Hospital, IRB in Tokyo Bay Urayasu Ichikawa Medical Center, IRB in Toyama University Hospital, IRB in Tokai University, IRB in St. Marianna University School of Medicine, IRB in Tsukuba Medical Center Hospital, IRB in Mitsui Memorial Hospital, IRB in Kinki University, IRB in Saiseikai Utsunomiya Hospital, IRB in IMS Tokyo Katsushika General Hospital, IRB in Nippon Medical School, Tokyo. All patients provided written informed consent for participation. This study was conducted in compliance with the principles of the Declaration of Helsinki. Additionally, the study was registered with the University Hospital Medical Information Network Clinical Trial Registry and accepted by the International Committee of Medical Journal (UMINID:000020423, the registry data from 14/03/2014).

The present analysis included consecutive patients who underwent TAVI with S3UR between March 2023 and September 2023 and a historical cohort of patients who underwent TAVI with S3 between September 2021 and September 2023 at the participating institutes. Patients who underwent TAVI for degenerated surgical bioprostheses or THV were excluded.

### Data collection and endpoint definitions

Baseline clinical, procedural, and follow-up data were prospectively recorded in a web-based database at each participating center. Regular follow-ups were scheduled pre-procedure, within 30 days after TAVI, and 1 year post-TAVI. Comprehensive clinical follow-up data were collected from medical records at each center, documentation from referring physicians, and/or telephone interviews. The web-based database was systematically audited by data committee members for completeness and accuracy to ensure data integrity. In cases of incomplete or inaccurate data, all participating centers were required to respond to queries.

Transthoracic echocardiography was performed before TAVI, at discharge, and annually thereafter. In accordance with the updated Valve Academic Research Consortium-3 (VARC-3) criteria^20^, prosthesis-patient mismatch (PPM) was assessed as the prosthesis effective orifice area (EOA) indexed to the body surface area and categorized as severe (≤0.65 cm^2^/m^2^ in the population without obesity [body mass index <30 kg/m^2^]; ≤0.55 cm^2^/m^2^ in the population with obesity [body mass index ≥30 kg/m^2^]) or moderate (>0.65 to 0.85 cm^2^/m^2^ in the population without obesity; >0.55 to 0.70 cm^2^/m^2^ in the population with obesity). All procedural complications were defined according to the VARC-3 criteria and relevant data were systematically collected. The clinical endpoints included all-cause mortality, stroke, and heart failure rehospitalization (HFH). Additionally, the incidence of aortic valve re-intervention was evaluated. A small annulus area was defined as an aortic annulus area ≤430 mm^2^, measured by multidetector computed tomography, based on the definition set by the pivotal SMART (SMall Annuli Randomized To EvolutTM or SAPIENTM Trial) ^21^.

### Statistical analysis

Categorical variables are reported as frequencies and percentages, with differences evaluated using the chi-square test or two-tailed Fisher’s exact test. Continuous variables are presented as means ± standard deviations or medians and interquartile ranges, with between-group comparisons performed using the two-sample t-test or the Mann–Whitney *U* test, as appropriate. For within-group comparisons, means and standard deviations were analyzed using a paired t-test. Time-to-event curves were depicted using the Kaplan–Meier method (censored at death or last valid contact in cases of those awaiting the next follow-up or consent withdrawal). A Cox proportional hazard model was used to calculate hazard ratios (HRs) and 95% confidence intervals (CIs) for clinical endpoints. All p-values were two-sided, and a *p* < 0.05 was considered significant for all tests.

Herein, we used propensity score matching to control for confounding factors due to baseline differences between patients undergoing TAVI with S3UR and S3. The propensity score was calculated using a multivariate logistic regression model based on the following 28 variables that could have affected study outcomes: age; sex; body mass index; Society of Thoracic Surgeons Predicted Risk of Mortality; clinical frailty scale score ≥4; New York Heart Association (NYHA) heart failure symptoms III or IV; hypertension; diabetes mellitus; peripheral artery disease; dialysis; chronic obstructive pulmonary disease; atrial fibrillation; hemoglobin level (g/dL); history of coronary artery disease; history of stroke; previous pacemaker implantation; echocardiographic variables such as aortic valve area (cm^2^), aortic valve mean pressure gradient (mPG) (mmHg), left ventricular ejection fraction (≥50%: preserved; ≥40% to <50%: mid-range; <40%: reduced), moderate or severe aortic regurgitation, moderate or severe mitral regurgitation, and moderate or severe tricuspid regurgitation; computed tomography variables such as annulus area (mm^2^), annulus perimeter (mm); and procedural variables such as access site (transfemoral approach or non), pre-dilatation, post-dilatation, and valve size (20 mm, 23 mm, 26 mm, 29 mm). Herein, a 1:1 greedy nearest neighbor matching protocol with a caliper of 0.2 was used. Absolute standardized differences were estimated for all baseline variables, and absolute standardized differences <0.10 were considered an indicator of good balance. The results for the overall matched cohort and cohorts comprising patients with small (20 and 23 mm) or large (26 and 29 mm) valve sizes were analyzed. In addition to valve size-based analyses, subgroup analyses were conducted based on annulus size, including small and large aortic annuli. SPSS version 29 (SPSS Inc., Chicago, IL, USA) was used for all statistical analyses.

## Supplementary information

Supplementary information

## Data Availability

The datasets generated and analyzed during the current study are not publicly available because OCEAEN-TAVI registry database is not for open access, but are available from the corresponding author on reasonable request.
