# Supplementary material for: Mechanisms underlying superior outcomes of transcatheter aortic valve implantation with the latest balloon expandable valve
Source: NPJ Cardiovasc Health. 2026 Mar 2;3:9. doi: 10.1038/s44325-026-00105-w (PMC12954083; doi:10.1038/s44325-026-00105-w)
Supplement: Supplementary file 1 — Supplementary information [file 44325_2026_105_MOESM1_ESM.pdf]

Figure S1. Kaplan–Meier curves comparing 1-year clinical outcomes for 20mm or 23mm S3UR and S3 valves.

a:

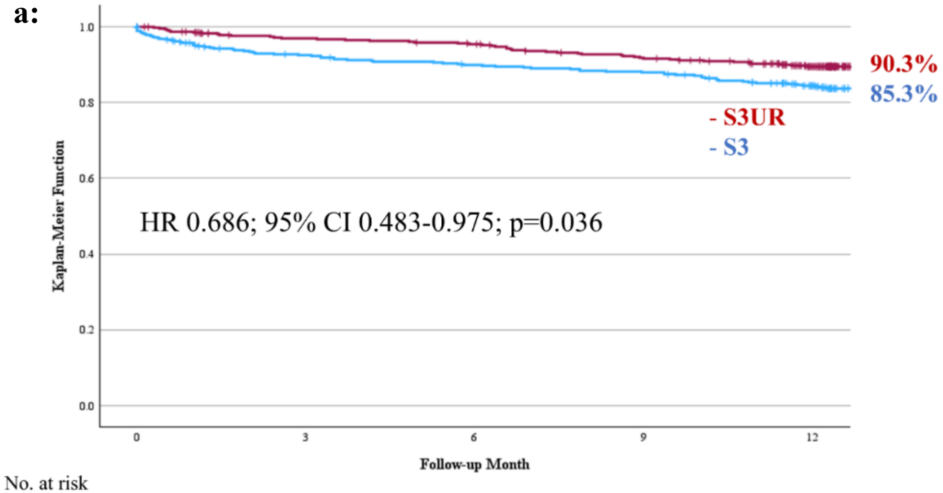

No. at risk					
S3UR	471	440	431	410	394
S3	477	427	413	404	387

b:

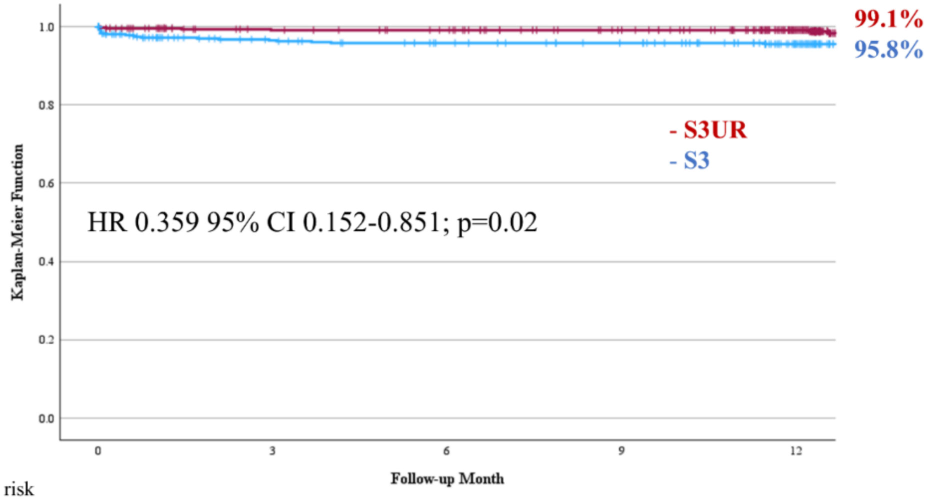

No. at risk					
S3UR	471	437	429	408	392
S3	477	418	404	395	380

c:

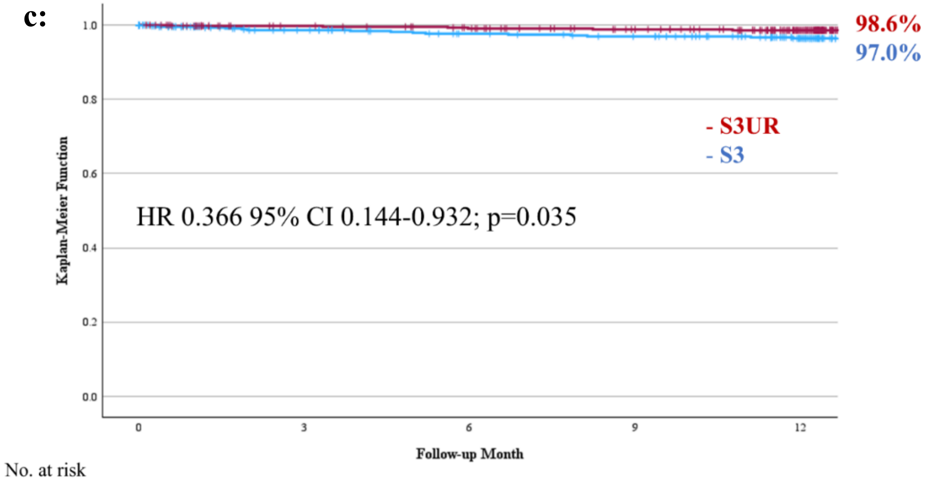

No. at risk					
S3UR	471	439	427	406	390
S3	477	423	405	396	379

(a) All-cause mortality (b) Stroke (c) Heart failure rehospitalization

S3UR, SAPIEN 3 Ultra RESILIA; S3, SAPIEN 3; HR, hazard ratio; CI, confidence interval.

Figure S2. Kaplan–Meier curves comparing 1-year clinical outcomes for 26mm or 29mm S3UR and S3 valves.

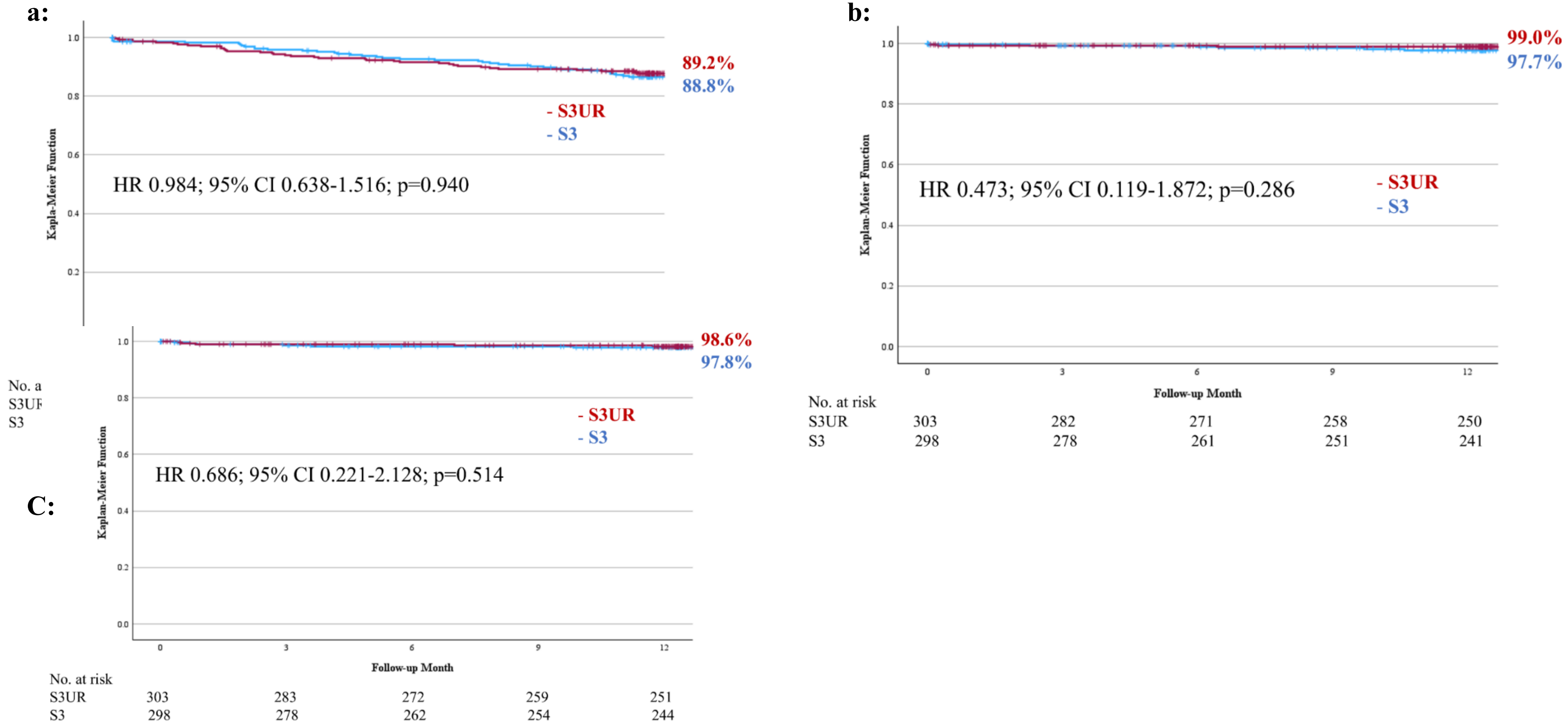

(a) All-cause mortality (b) Stroke (c) Heart failure rehospitalization

S3UR, SAPIEN 3 Ultra RESILIA; S3, SAPIEN 3; HR, hazard ratio; CI, confidence interval.

Table S1. Baseline characteristics of the matched cohort by small or large size

	Small (20mm or 23mm)			Large (26mm or 29mm)		
	**S3UR** N=471	**S3** N=477	p	**S3UR** N=304	**S3** N=298	p
Age, years	84.0±6.2	84.5±6.1	0.229	83.0±5.6	82.5±6.7	0.575
Sex (male)	89 (18.9)	86 (18.0)	0.731	231 (76.0)	228 (76.5)	0.88
Body mass index, kg/m²	22.3±4.2	22.3±4.0	0.491	22.5±3.4	22.6±3.8	0.842
STS-PROM score, %	6.2 [4.0-9.5]	6.1 [4.1-9.6]	0.646	5.7 [3.8-8.6]	4.9 [3.3-8.6]	0.104
Clinical frailty scale>=4	115 (24.4)	130 (27.3)	0.077	60 (19.7)	58 (19.5)	0.371
NYHA III or IV	129 (27.4)	163 (34.2)	0.024	104 (34.2)	90 (30.2)	0.293
Hemoglobin, g/dL	11.4 [10.4-12.8]	11.4 [10.4-12.6]	0.705	11.6 [10.5-12.8]	11.6 [10.2-12.8]	0.392
Brain Natrium Peptide, pg/ml	160 [71-490]	274 [112-685]	<0.001	270 [100-714]	263 [103-728]	0.595
**Concomitant diseases**						
Hypertension	382 (81.1)	402 (84.3)	0.197	244 (80.3)	246 (82.6)	0.471
Diabetes mellitus	156 (33.1)	427 (26.6)	0.029	95 (31.3)	111 (37.2)	0.121
Dyslipidemia	251 (53.3)	240 (50.3)	0.359	162 (53.3)	150 (50.3)	0.475
Chronic kidney disease (GFR<60mL/min/1.73m²)	359 (76.2)	360 (75.5)	0.788	223 (73.4)	226 (75.8)	0.484
Dialysis	80 (17.0)	98 (20.5)	0.161	86 (28.3)	79 (26.5)	0.625
COPD	35 (7.4)	32 (6.7)	0.664	32 (10.5)	35 (11.7)	0.635
Atrial fibrillation	77 (16.3)	102 (21.4)	0.048	82 (27.0)	74 (24.8)	0.549

Coronary artery disease	138 (29.3)	150 (31.4)	0.472	124 (40.8)	113 (37.9)	0.471
Peripheral artery disease	66 (14.0)	62 (13.0)	0.648	53 (17.4)	59 (19.8)	0.456
Carotid stenosis	25 (5.3)	18 (3.8)	0.314	22 (7.2)	22 (7.4)	0.945
**Previous history**						
Smoking	78 (16.6)	89 (18.7)	0.451	135 (44.4)	153 (51.3)	0.155
History of PCI	65 (13.8)	94 (19.7)	0.030	78 (25.7)	73 (24.5)	0.742
History of CABG	13 (2.8)	12 (2.5)	0.814	15 (4.9)	10 (3.4)	0.332
History of myocardial infarction	17 (3.6)	27 (5.7)	0.133	26 (8.6)	28 (9.4)	0.717
History of stroke	42 (8.9)	51 (10.7)	0.399	43 (14.1)	39 (13.1)	0.705
Previous pacemaker	27 (5.7)	23 (4.8)	0.531	13 (4.3)	18 (6.0)	0.328
**Echocardiographic data**						
Aortic valve area, cm²	0.67±0.19	0.66±0.20	0.14	0.73±0.19	0.73±0.18	0.941
Index aortic valve area, cm²/m²	0.47±0.14	0.46±0.14	0.162	0.46±0.12	0.46±0.12	0.62
Peak flow velocity, m/s	4.4±0.64	4.3±0.70	0.504	4.1±0.62	4.2±0.64	0.094
Aortic valve peak gradient, mmHg	78.3±23.2	77.3±24.6	0.504	68.5±20.5	70.7±21.7	0.128
Aortic valve mean gradient, mmHg	45.6±14.8	44.8±15.2	0.61	39.4±12.8	41.2±13.4	0.029
Left ventricular ejection fraction, %	62.9±10.1	61.5±11.2	0.044	54.0±13.5	55.6±13.1	0.097
Preserved (>=50%)	420 (89.2)	420 (88.1)	0.177	203 (66.8)	210 (70.5)	0.414
Mid-range (>=40, <50%)	32 (6.8)	26 (5.5)		45 (14.8)	45 (15.1)	
Reduced (<40%)	19 (4.0%)	31 (6.5)		56 (18.4)	43 (14.4)	
Aortic regurgitation ≥moderate	43 (9.1)	43 (9.0)	0.951	43 (14.1)	43 (14.4)	0.92

Mitral regurgitation ≥moderate	55 (11.7)	64 (13.4)	0.419	36 (11.8)	31 (10.4)	0.574
Tricuspid regurgitation ≥moderate	31 (6.6)	34 (7.1)	0.739	19 (6.3)	22 (7.4)	0.581
**Computed Tomography**						
Annulus Area, mm^2	375±39	374±40	0.994	497±57	495±63	0.734
Area ≤430	436 (92.6)	449 (94.1)	0.335	20 (6.6)	39 (13.1)	0.007
Perimeter, mm	70.2±3.7	70.0±3.7	0.625	80.5±4.8	80.4±4.9	0.621

Depicted are means with standard deviations (±SD), or counts with percentages (%). S3UR, SAPIEN 3 Ultra RESILIA; S3, SAPIEN 3; STS-PROM, Society of Thoracic Surgeons-predicted risk of mortality; NYHA, New York Heart Association; GFR, Glomerular Filtration Rate; COPD, Chronic Obstructive Pulmonary Disease; PCI, percutaneous coronary intervention; CABG, coronary artery bypass grafting; ASD, absolute standardized difference. P-values from Fisher's test (2 x 2 comparison), chi-square test (n x 2 comparisons) or t-tests (continuous parameters).
